# Defining Cell Identity with Single‐Cell Omics

**DOI:** 10.1002/pmic.201700312

**Published:** 2018-05-28

**Authors:** Laura Mincarelli, Ashleigh Lister, James Lipscombe, Iain C. Macaulay

**Affiliations:** ^1^ Earlham Institute Norwich Research Park Norwich NR4 7UZ United Kingdom

**Keywords:** epigenomics, genomics, proteomics, single‐cell, technology, transcriptomics

## Abstract

Cells are a fundamental unit of life, and the ability to study the phenotypes and behaviors of individual cells is crucial to understanding the workings of complex biological systems. Cell phenotypes (epigenomic, transcriptomic, proteomic, and metabolomic) exhibit dramatic heterogeneity between and within the different cell types and states underlying cellular functional diversity. Cell genotypes can also display heterogeneity throughout an organism, in the form of somatic genetic variation—most notably in the emergence and evolution of tumors. Recent technical advances in single‐cell isolation and the development of omics approaches sensitive enough to reveal these aspects of cell identity have enabled a revolution in the study of multicellular systems. In this review, we discuss the technologies available to resolve the genomes, epigenomes, transcriptomes, proteomes, and metabolomes of single cells from a wide variety of living systems.

## 1 Introduction

All living systems, from bacterial populations to complex multi‐cellular organisms, are composed of communities of individual cells. The cell is thus a fundamental unit of biology, and the capacity to analyze the behavior of organs and organisms at the single‐cell level is critical to developing and understanding of the emergent behaviors of these communities of cells.

Increased biological complexity is enabled by the ability of cells to differentiate and attain distinct “identities” within a system—reflecting a divergence in form or function from precursor cells. This identity has largely been defined in terms of cell type and cell state. Precise definitions for these terms remain elusive: cell type has historically been described by observing reproducible functional distinctions in vivo or in vitro (often coupled with expression of a set of marker genes), while cell state refers to dynamic, responsive changes that alter the phenotype and function of the cell, but not so significantly that a new cell type is acquired.

**Figure 1:**
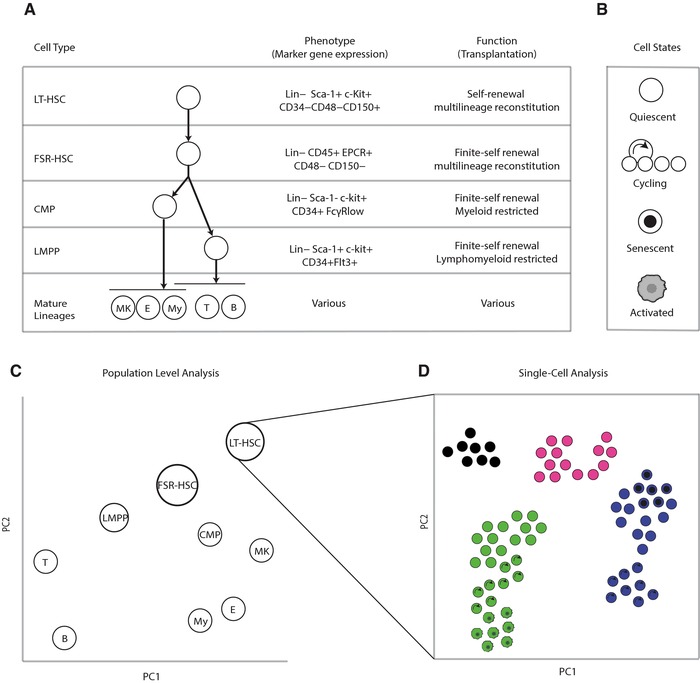
Definitions of cell type and state. A) In the hematopoietic system, cell types have been typically defined by a combination of cell surface marker expression and functional output in in vitro and in vivo assays. B) Within cell types, multiple cell states are possible, including quiescence, active cycling, senescence, and in some cases, resting and activated states. C) Population‐level characterization enables molecular definition of the differences between cell types, in this hypothetical example using principal components analysis (PCA) of RNA‐seq data. This, however, does not reveal heterogeneity within these phenotypically defined populations. Through (D) single‐cell analysis, it is possible to explore this heterogeneity, even in rare cell populations such as HSCs, revealing novel cell phenotypes—cell types and states—within a “homogeneous” population of cells. Abbreviations: LT‐HSC, long‐term reconstituting HSC; FSR‐HSC, finite self‐renewal HSC; LMPP, lymphoid‐primed multipotential progenitors; CMP, common myeloid progenitor; MK, megakaryocyte; E, eryrthroid; My, Myeloid; T, T‐cell; B, B‐cell; PC, principal component.

As an example, the murine hematopoietic system (Figure 1) consists of a number of well‐defined cell types, characterized by functional behavior in standardized assays. The hematopoietic stem cell (HSC), the cell type which resides at the apex of the hematopoietic hierarchy, has widely accepted functional and phenotypic definitions. Functionally, this cell type is defined by the ability to reconstitute all mature blood cell lineages after transplantation (multilineage potential) and to do so for long periods, including after serial transplantation, through the generation of new HSCs (self‐renewal).^1^ A variety of cell surface markers have been used to define stem cell phenotypes with increasingly high functional purity, and in some cases with the potential to further classify functional subtypes of HSCs.^2^, ^3^

HSCs must enter into different states to fulfil their functional roles. The hematopoietic system is highly dynamic and responsive, and at all levels, changes in cell state are possible—and indeed essential—HSCs are typically quiescent, but must enter a cell cycle “state” to produce new progeny to maintain the blood supply and the stem cell pool. Since this pool is maintained, cell type is based on cell‐intrinsic properties that can be passed on to daughter cells during mitosis; however, since HSCs are pluripotent, cell types can enter into intermediate states as they transition into committed and mature cell lineages.

Cell type and state, as defined in this example, are a product of the cell's molecular profile—including genomic, epigenomic, transcriptomic, proteomic, and metabolomic aspects—which in turn emerge from cell intrinsic and extrinsic factors. The recent development of approaches sensitive enough to assess the molecular profiles of individual cells offers the opportunity to gain a new perspective on our definitions of cell identity, and to more clearly delineate the processes by which a single stem cell can generate an entire hematopoietic system, or indeed how a complex multicellular organism can arise from a single zygotic cell.

## 2 Concepts and Methods for Single‐Cell Isolation and Profiling

**Figure 2:**
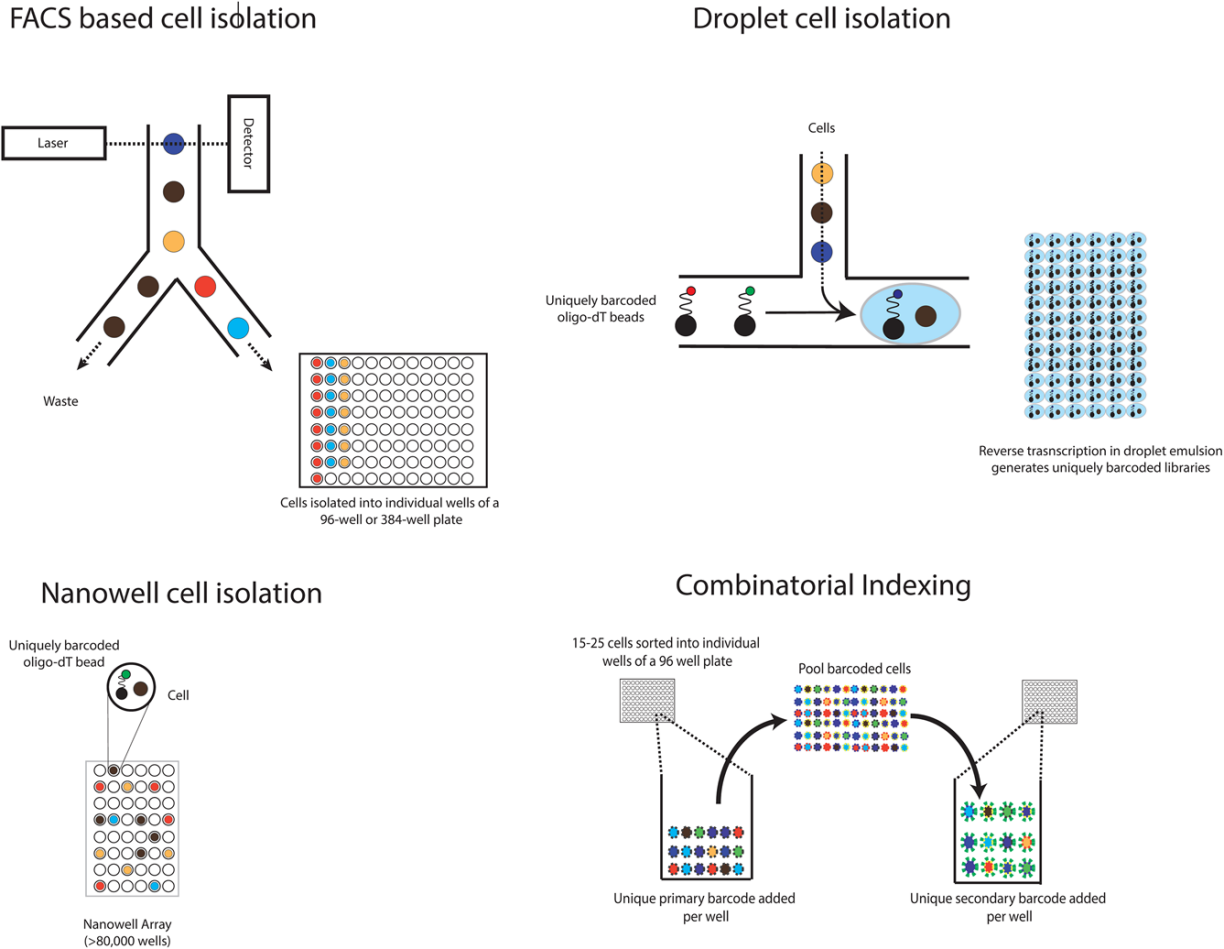
Methods for isolation and sequencing of single cells. A) FACS‐based cell isolation enables selective deposition of single cells into multiwell plates for downstream molecular processing. Index sorting allows some information about each cell's phenotype to be recorded as it is deposited into the well. Once the cells have been deposited, a number of molecular processes are possible. B) Droplet‐based cell isolation involves the partitioning of single cells into individual droplets with uniquely barcoded oligonucleotides. In the case of single‐cell mRNA‐seq these barcoded oligos prime first strand synthesis of cDNA from the poly‐A tail. Reverse transcription is then performed in a droplet emulsion, resulting in each cDNA molecule being uniquely tagged based on its cell of origin. Unique molecular identifiers (UMIs) are also incorporated to enable unequivocal counting of the number of detected molecules. C) Nanowell‐based approaches use a similar approach, but rather than partitioning cells into droplets, cells are captured in minute wells with uniquely barcoded beads. D) Combinatorial indexing strategies have used a two‐step barcoding strategy for DNA or cDNA molecules to increase throughput without the need for microfluidics. First, a primary barcode is added to small pools of FACS isolated cells/nuclei (in the case of cDNA, this is added during reverse transcription, in the case of DNA this is added through tagmentation with barcoded adaptors) which are then re‐pooled with other distinctly barcoded cells and again sorted into small pools, where they received a second barcode. Thus, each cell receives a unique pairing of barcoded molecules, enabling each sequencing read to be assigned to an individual cell.

Key to the study of single cells is the capacity to effectively isolate them to enable analysis of the cell's unique molecular identity, and there are numerous methods for doing so (Figure 2). While manual isolation of cells, using micropipettes or micromanipulation is feasible; the throughput is too low to permit broad studies of cellular heterogeneity. However, these remain the only methods by which the biopsy of daughter cells from a single‐cell division^4^ can be performed. Flow cytometry has enabled analysis of small panels of proteins/markers in individual single cells, and fluorescence‐activated cell sorting (FACS)–based isolation has long been employed for the functional and molecular profiling of heterogeneous cell populations.

**Figure 3:**
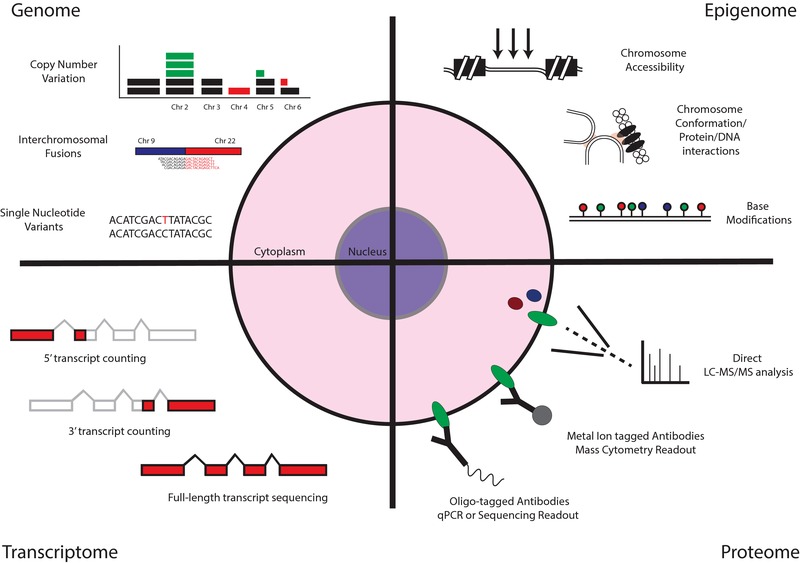
Methods for the analysis of single‐cell identity. An overview of the methods currently available to study the genome, epigenome, transcriptome and proteomes of single cells, some of which have been combined into multi‐omic single‐cell assays.

Demand for higher throughput—from hundreds to millions of cells—has driven the development of microfluidic approaches for cell isolation (reviewed in detail in ref. 5), in which cells are captured in individual droplets or nanowells for processing, thus maximizing throughput and minimizing the reagent cost per cell. Where the isolation of single cells from tissues has proved challenging, for example in primary tissue, nuclear isolation has also been demonstrated to be a successful approach for the analysis of cellular genomes^6^, ^7^ and transcriptomes,^8^, ^9^, ^10^ compatible with both FACS‐based and droplet‐based approaches.

Most recently, combinatorial indexing strategies have been employed to generate genomic,^11^ epigenomic,^12^ and transcriptomic^13^ libraries from mammalian single cells, in experiments which analyze greater than 10 000 cells in parallel. Combinatorial indexing involves barcoding pools of single cells with one of 384 barcodes using either a barcoded reverse transcription primer for RNA libraries or transposase mediated barcoding for DNA libraries (Figure 2). These 384 reactions are then pooled and 15–25 single cells from the pool are FACS‐sorted into each well of a new 96‐well plate and a second barcode is added by PCR. Each well will then contain a pool of virtually uniquely barcoded libraries, having received one barcode in the first pool and subsequently a second in the second pool.

All current Next Generation Sequencing (NGS) platforms require that the minute amounts of DNA or RNA present within a single cell be amplified to generate sufficient material to sequence. Due to the accessibility of approaches to replicate nucleic acids in vitro, a multitude of methods have emerged to perform this amplification on single‐cell genomes, epigenomes, and transcriptomes, while the embryonic field of single‐cell proteomics relies on the development of methods which either translate protein abundance into amplifiable nucleic acid signatures or the direct measurement of protein abundance by sensitive mass spectrometry (Figure 3).

### 2.1 Single‐Cell Genomics

It is widely thought that the genome is relatively stable throughout life and remains the same independently of cell type—with the exception of post‐meiotic germ cells, vertebrate immune cells, and in pathological conditions such as cancer. However, during the generation of maintenance of the extraordinary number of cells that comprise a complex multicellular organism—in adult humans, the total cell number is estimated to be 38 trillion^14^ at any given time, with a substantial ongoing turnover of particular cell lineages. There is therefore considerable opportunity for the acquisition of somatic variation—mutations that will be distinct between cells from the same organism, only a fraction of which will be pathogenic.

Genomic heterogeneity between individual cells of the same organism can occur on multiple scales, including single nucleotide variants (SNVs), indels, inter‐ and intra‐chromosomal fusions, copy number variants (CNVs) and aneuploidies of whole chromosomes, as well as insertion sites of viruses or mobile elements (e.g., L1 retrotransposition events). By sequencing the genomes of single cells, each of these phenomena can be studied with greater resolution than by sequencing a “bulk” DNA specimen. Since these events are acquired through cell division and errors in DNA replication, it is possible to use patterns of somatic variation to reveal cell lineages in both normal and diseased tissues, by examining the sequential acquisition of particular mutations,^7^ which will have particular implications in the study of clonal evolution of cell lineages in pre‐cancerous conditions and cancer.

Several approaches to amplify single‐cell DNA have been applied, each with advantages and disadvantages (Table 1). The technical limitations and advantages of these methods have been described in detail elsewhere;^15^ however, it is important to note that none of these methods are perfect, and obtaining base‐level resolution with broad‐coverage of single‐cell genomes remains a challenge. Common challenges include allele and/or locus dropout, biased amplification of GC‐rich sequences^16^ and the generation of chimeric products,^17^ all which can confound effort to accurately sequence and assemble single‐cell genomes. Base‐level analysis of a single‐cell's genome is also costly, as achieving accurate base calling requires significant coverage per cell, thus much of the current literature focusses on the analysis of CNVs in mammalian cells.

**Table 1:** Methods for whole‐genome amplification and sequencing of DNA from single cells

Method	Platform	Number of Cells (typical)	Description	Advantages	Disadvantages	Applications
**Multiple displacement amplification (MDA)** 17, 18	Microwell plate/tubes	10s–100s	Randomly primed isothermal amplification of DNA by Phi29 polymerase	Broad genome coverage High fidelity amplification (Error rate 1.2 × 10–5)	Potential for inaccurate identification of CNVs due to allelic drop out, GC bias and uneven amplification Generation of chimeric molecules	Genome‐wide or targeted SNV and structural variant analysis WGA‐X uses a thermostable version of phi 29 called Equiphi 2 for the amplification of DNA from microbial cells or cells from environmental samples^115^ eMDA (emulsion multiple displacement amplification) can be used to call SNVs^118^
**PicoPLEX (Rubicon Genomics)**	Microwell plate/tubes	10s–100s	Two‐stage (linear and exponential) amplification of DNA	Accurate representations of CNVs	Higher error rate than MDA, potential for inaccurate SNV calling	Genome‐wide CNV analysis
**MALBAC** 21 **(Yikon Genomics)**	Microwell plate/tubes	10s–100s	Two‐stage (linear and exponential) amplification of DNA	Broad genome coverage (85–93%) Accurate representations of CNVs Lower allelic dropout than MDA or PicoPLEX	Higher error rate than MDA	Genome‐wide CNV analysis Pre‐implantation genetic screening of genomic or mitochondrial DNA (Shang et al., 2017)
**AMPLI1 (Silicon Biosystems)**	Microwell plate/tubes	10s–100s	DNA digestion and adaptor ligation, PCR amplification	Broad genome coverage Lower allelic dropout than MDA, PicoPLEX and MALBAC	Less efficient enrichment in targeted genome sequencing than other methods	Genome‐wide CNV or SNV detection
**Strand seq** 119	Microwell plate/tubes	10s–100s	Tagging of individual DNA strands during replication using BrdU followed by selective depletion of tagged strands Direct library preparation from BrdU‐ strands	Generates sequences from homologous chromosomes Direct library construction without WGA, reduced sequence bias and allelic drop‐out	Requires that the sample can be treated with BrdU for one round of cell division—difficult to apply in vivo	Detection of copy neutral genomic rearrangements Studying meiotic recombination Studying inheritance‐ by haplotyping/ phasing
**SCI‐seq** 11	Combinatorial indexing	1000s–10 000s	Dual indexing of individual nucleosome depleted genomes Index 1 is added by tagmentation of 96 pools of 2000 nuclei each Tagged cells are pooled and re‐sorted, with Index 2 added by PCR amplification of pools of 22 cells	High throughput No custom equipment required, uses FACS for isolation and pooling of nuclei	Shallow sequencing of individual cells therefore only large CNVs can be analyzed	CNV analysis in large numbers of single cells
**SiC‐seq** 22	Custom microfluidics	1000s–10,000s	Single‐cell encapsulation in droplets with uniquely barcoded adaptors	High throughput	Shallow sequencing of individual cells, only small (microbial) genomes have been analyzed to date Custom microfluidics required	Analysis of microbial populations at single‐cell resolution In theory can be applied to eukaryotic cells for CNV analysis
**SISSOR (single‐stranded sequencing using microfluidic reactors** 120	Custom microfluidics	10s	Separate the Watson and Crick DNA strands; randomly partition megabase‐size fragments into multiple nanoliter compartments for amplification and construction of barcoded libraries for sequencing	Low error rate (1 × 10–8) Ability to assemble large fragments of single‐cell genomes (N50 > 7 Mb, largest contig 9 Mb)	Low throughput—only tested on three human cells, though has potential for high‐throughput modifications	SNV calling in single cells Single‐cell genome assembly and long read, haplotyping information such as HLA haplotyping for donor‐patient matching
**MIDAS (microwell displacement amplification system)** 19	Custom nanowell array	100s–1000s	Cells are captured in nanowells and MDA performed in ∼12 nL volumes Successful reactions are then picked for sequencing	Less amplification bias compared to conventional MDA More even coverage than MDA Lower reaction volumes reduce cost Demonstrated to work with microbial and human cells	No commercial availability of the microwell arrays Low cell loading numbers Micromanipulation used to aspirate amplified DNA from microwells	CNV analysis in single cells

Multiple displacement amplification (MDA)–based methods remain the most applicable where base‐level events (e.g., SNVs) are to be investigated, as the phi29 enzyme utilized in the amplification has an extremely low error rate.^18^ However, these methods are less appropriate for CNV analysis as the coverage of the genome is generally uneven and thus generate “noisy” copy number profiles, although there is evidence that when performed in microfluidic chambers or nanowells,^19^ this effect can be mitigated. Nevertheless, when CNVs are of interest, degenerate oligonucleotide PCR (DOP‐PCR^20^)–based quasi‐linear approaches such as PicoPlex (Rubicon Genomics) and MALBAC^21^—which more accurately preserve copy number—are generally preferable.

The vast majority of single‐cell genome sequencing has explored only tens to hundreds of cells, but recent developments have applied combinatorial indexing^11^ and microfluidic droplet barcoding^22^ to enable 10 000s of cells to be processed in parallel. Current NGS capabilities do not extend to base‐level analysis of these numbers of mammalian cells, and these approaches are best applied to CNV analysis in organisms with larger genomes. These approaches are particularly important for the analysis of microbial communities, where the small genome size will enable affordable sequencing of large numbers of individual cells.

### 2.2 Single‐Cell Epigenomics

Epigenomic aspects of cell identity, including DNA methylation, histone modification, chromatin accessibility, and chromosome conformation, are dynamic, regulatory mechanisms that enable cells with identical genotypes to have distinct gene expression profiles. Thus, by representing the “functionalization” of the genome, these different mechanisms are key aspects of how cell type and state should be defined. Epigenomic diversity is also often heritable through cell division, and as such, offers a mechanism by which cells within a specific type can proliferate and maintain their identity. There has been a substantial proliferation in the diversity of epigenetic techniques aimed to study single cells (reviewed in detail in ref. 23)—it is now possible to survey a majority of known epigenetic aspects of a single cell with varying degrees of resolution and throughput (Table 2).

**Table 2:** Methods for epigenomic analysis of single cells

Modification	Method	Platform	Number of Cells (typical)	Description
**DNA methylation**	ScBS‐seq^24^	Microwell plate/tubes	10–100s	Bisulfite conversion of unmodified C to T, 5mC remains unconverted
	scRRBS^121^	Microwell plate/tubes	10–100s	Reduced representation bisulfite sequencing, enables single‐base resolution DNA methylation
	scAba‐seq^28^	Microwell plate/tubes	10–100s	Hydroxymethylation (5hmC) profiling. Glucosylation of 5hmc position generates recognition sites for restriction endonuclease AbaSI
	CLEVER‐seq^29^	Microwell plate/tubes	10–100s	Formylcytosine (5fC) detection by direct chemical labeling with malonitrile. Subsequent conversion C‐T at 5fC labeled sites during amplification and sequencing
**Chromatin accessibility**	NOME‐seq^34^	Microwell plate/tubes	10–100s	Methyltransferase (methylase) enzyme is used to label accessible (or nucleosome depleted) DNA prior to bisulfite sequencing which distinguishes between methylated and unmethylated chromatin states
	scDNAse‐seq^31^	Microwell plate/tubes	<1000	Method of detecting genome‐wide DHSs, DNase I hypersensitive sites. This technique enables genome‐wide mapping of hypersensitive site, therefore of active regulatory elements of transcription
	scATAC‐seq^32^, ^33^	Fluidigm C1 platform	10–100s	Individual cells are captured and assayed on Fluidigm platform. Tn5 transposase tags regulatory regions by inserting sequencing adapters into accessible regions of the genome, allowing measurement of open chromatin sites
	scATAC‐seq^12^	Combinatorial indexing	10 000s	Integration of combinatorial cellular indexing and ATAC‐seq to measure chromatin accessibility in large numbers of single cells
**Histone modifications**	scChiP‐seq (drop‐Chip)^35^	Custom microfluidics platform	1000s–10 000s	Droplet‐based microfluidic system process single cell to indexed chromatin fragments. Indexed chromatin from multiple cells can then be combined and subsequently immunoprecipitation can be performed
**Chromosome organization**	scDam‐ID^37^	Microwell plate/tubes	100	Enables mapping of genome‐wide nuclear lamina interactions domains in single human cell. Dam adenosine transferase methyltransferase is fused with lamin B1 (constituent of nuclear lamina) and expressed in cells so that sites interactions are mapped from sequence tags after DpnI digestion
	scHi‐C^40^	Microwell plate/tubes	10–100s	Identifies DNA sequences in close spatial proximity in the nucleus after restriction enzyme digestion and DNA ligation
	scHi‐C^39^	Microwell plate/tubes	10–100s	Improved Hi‐C protocol able to determine whole‐genome structures of single G1‐phase haploid cells and define 3D models of chromosome organization

Modifications of DNA, including cytosine methylation (5mC), hydroxymethylation (5hmC) and formylcytosine (5fC) can regulate gene expression in the short and long term, with 5mC associated with transcriptional repression, while 5hmC and 5fC are associated with transcriptional activation. Assays to detect each of these modifications across the genomes of single‐cells have been developed, though they still present several technical challenges. Single‐cell bisulfite sequencing (scBS‐seq),^24^, ^25^ which detects 5mC, is powerful because it allows assessment of a large fraction of promoters with relatively low sequencing costs, but its limitation is poor coverage—only 20–40% of the genome, including many important regulatory regions, are sequenced due to the destructive nature of bisulfite treatment. Other potential drawbacks are low mapability (about 30%) and high level of PCR duplicates.^24^

Overall, this sparseness of data limits the analysis of cellular variation and thus it is necessary to use methylation frequency data from single‐cell populations^26^ or approaches to impute missing values.^27^ The recently described single‐cell 5hmC sequencing (scAba‐seq),^28^ which uses the restriction endonuclease AbaSI to induce double‐strand breaks in modified DNA sequences, followed by adaptor ligation, amplification, and library prep and single‐cell 5fC sequencing (chemical‐labeling‐enabled C‐to‐T conversion sequencing, CLEVER‐seq)^29^ have been applied to generate low‐coverage modification profiling of single‐cell genomes. Similar to scBS‐seq approaches, these low‐coverage approaches have not yet demonstrated genome‐wide base‐level modification, although single‐base resolution with incomplete genome coverage was demonstrated with CLEVER‐seq.^29^

Chromatin accessibility, which is a regional marker of “activity” within the genome, can be assessed through treatment of permeabilized cells or nuclei with enzymes which cleave at sites where DNA is not protected by chromatin. Single‐cell DNAse‐seq^30^, ^31^ digests open DNAse I hypersensitive sites (DHS) throughout the genome and single‐cell transposase‐accessible chromatin sequencing (scATAC‐seq) uses the Tn5 transposase to directly insert sequencing library adaptors into open chromatin regions prior to PCR‐based amplification of the tagmented regions.^12^, ^32^, ^33^ Single‐cell nucleosome occupancy and methylome‐sequencing (scNOMe‐seq)^34^ uses a GpC methyltransferase (MTase) step, followed by bisulfite treatment and NGS to simultaneously measure chromatin accessibility (through GpC methylation) and endogenous CpG methylation. Combinatorial indexing approaches have also been applied to scATAC‐seq, enabling high‐throughput shallow profiling of the chromatin states of thousands of single cells.^12^

Single‐cell droplet–based chromatin immunoprecipitation (drop‐ChIP) has been demonstrated, although using extremely sparse data (1000 reads per cell) to generate population distributions of histone 3 lysine 4 trimethylation (H3K4me3), enabling detection of discrete epigenetic states in populations of mouse ES cells, fibroblasts, and a hematopoietic cell line.^35^ As such, genome‐wide DNA‐protein interactions in single cells has yet to be demonstrated, though this would offer a powerful tool to assess heterogeneity in regulation of gene expression.

DNA/laminin associations can be probed using the Dam‐ID protocol^36^ which has been adapted to work with single cells. Using a haploid cell line (KBM7) engineered to express a DNA adenine methyltransferase (Dam)/Lamin B1 (LmnB1) fusion protein to modify adenine bases that are in physical proximity to laminin proteins in nuclear membrane, enabling mapping of lamina‐associated domains (LADs), which may offer an insight into cell state and the regulation of gene expression, as most genes in LADs are expressed at low levels.^37^ However, as this method currently requires genetic manipulation, and is ideally performed in a haploid cell line to enable unambiguous chromosomal identification, this method is unlikely to immediately applicable to more complex systems. Single‐cell Hi‐C, which measures the spatial proximity of regions of DNA, has also been demonstrated in haploid cells.^38^, ^39^, ^40^

As with single‐cell genome analysis, each of these epigenetic methods tend to generate sparse data which preclude true “genome‐wide” identification of DNA modifications, open chromatin sites, and chromatin conformation, and while imputation approaches are available to complete missing information,^25^, ^32^ it is important to note that no method exists for robust base‐level resolution genome‐wide epigenomic profiling of a single cell. Consideration of the biological sources of variation is also essential in single‐cell epigenomics experiments as some cellular features, such as cell cycle, are dominant drivers of gene expression^41^ and chromatin accessibility variation^38^ in single cells.

### 2.3 Single‐Cell Transcriptomics

The transcriptome is highly dynamic, reflecting cell type and cell state. Cells committed to particular type would be expected to express selections of marker genes which differentiate them from other cells within the population, and within each type, variation in gene expression may be indicative of cell state transitions—for example, specific gene expression profiles can be associated with cell cycle status.^41^, ^42^

Perhaps the greatest technical and experimental advances using single‐cell techniques have been in the study of the transcriptome. This is largely due to the accessibility of the transcriptome, which in contrast to the genome is present at relatively high copy number per cells—transcript copy numbers can range from 1–100 000 copies per transcript per cell, although the majority are estimated to be present at less than 100 copies per cell.^43^ In eukaryotic systems, naturally occurring polyadenylation of the 3′ tail of mRNA molecules offers a near‐universal priming site to generate first‐strand cDNA, which through ligation or incorporation of adaptor sequences can act as a template for linear amplification using a multitude of methods (Table 3) including specially adapted in vitro transcription (IVT) protocols^44^ or PCR‐based protocols.^45^, ^46^, ^47^

**Table 3:** Methods for transcriptomic analysis of single cells

Method	Platform	Number of Cells (typical)	Description	UMI	Applications	Typical number of sequencing reads per cell
Smart‐seq/Smart‐seq2^46^	Microwell plate/tubes/Fluidigm C1 platform	100s–1000s	Template‐switching PCR‐based full‐length transcript amplification. Can be applied to cells or nuclei (scNuc‐seq)	No	Transcript enumeration Analysis of alternative splicing allelic expression	500 000–4 000 000
**CEL‐Seq/CEL‐Seq2** 44	Microwell plate/tubes	100s–1000s	In vitro transcription‐based 3′ transcript amplification	Yes	Transcript quantification	100 000–1 000 000
**STRT** 45, 52	Microwell plate/tubes (also modified for ICell8 Nanogrid^52^	100s–1000s	Template‐switching PCR‐based full‐length transcript amplification followed by 5′ selection	Yes	Transcript quantification	100 000–1 000 000
**sci‐RNA** 13	Combinatorial indexing	1000s–10 000s	Combinatorial indexing approach in which transcripts are first indexed during first strand synthesis and subsequently during PCR of 3′ sequencing tags	Yes	Transcript quantification	20 000–200 000
Droplet‐based approaches	**Microfluidic platforms**: Drop‐seq^49^ InDrops^48^ **Commercial Platforms**: 10X Genomics Chromium Dolomite Nadia	1000s–10 000s	Cells are partitioned into individual droplets and cDNA molecules are uniquely barcoded during reverse transcription	Yes	Transcript quantification	20 000–200 000
Nanowell approaches	**Custom Nanowell Chip** SeqWell^51^ **Commercial Platforms**: Nanogrid (ICell8) BD Rhapsody	1000s–10 000s	Cells are partitioned into individual wells of a custom built nanowell chip and cDNA molecules are uniquely barcoded during reverse transcription	Yes	Transcript quantification	20 000–200 000

While many single‐cell transcriptomics approaches can readily be applied in medium throughput on FACS‐sorted cells isolated into multi‐well plates, the scale and ease with which single‐cell transcriptomics experiments can be undertaken has benefited considerably from the introduction of combinatorial indexing,^13^ microfluidic^48^, ^49^, ^50^ and nanowell^51^, ^52^ approaches to partition and/or barcode individual cells.

Smart‐seq/Smart‐seq2, which enables full‐length amplification of single‐cell cDNA can readily be applied to FACS isolated cells in multi‐well plates^53^, ^54^ or cells isolated on Fluidigm's C1 microfluidic instrument.^55^ By combining full‐length, PCR‐based cDNA amplification with tagmentation‐based NGS library preparation, it is possible to enumerate transcript abundance within the cell, but also to explore sequence variation (SNVs, UTRs, and alternative splicing) within the transcriptome. This method has also been demonstrated to work successfully on nuclei from tissues where single‐cell isolation is challenging.^9^ We and others have also demonstrated that single‐cell libraries generated using this approach are compatible with long‐read sequencers (e.g., Pacific Biosciences and Oxford Nanopore technology),^4^, ^56^ enabling unequivocal identification of splice variants.

CEL‐seq/CEL‐seq2, which relies on IVT‐based amplification and 3′ enrichment, generates 3′‐end cDNA libraries, incorporating unique molecular identifiers (UMIs)—random barcodes added to the sequence during the first reverse transcription step.^44^ These subsequently enable quantification of unique mRNA molecules within the cell with higher accuracy than Smart‐seq2, which does not currently include UMIs and requires normalization by gene length to generate corrected expression values. Other tag‐sequencing methods have been developed, including 5′ STRT^45^, ^52^ and similarly incorporate UMIs for transcript counting.

High‐throughput single‐cell gene expression profiling has been revolutionized by the introduction of microfluidics approaches which enable 3′ transcript counting from thousands of cells in parallel. The Drop‐seq^49^ and Indrop^50^ methods introduced this approach, in which individual cells or nuclei^10^ are co‐encapsulated in droplets with uniquely barcoded oligo‐dT primers, enabling cDNA to be pooled and sequenced in parallel, with reads assigned to individual cells based on their barcode. Droplet‐based methods have also been integrated with pooled CRISPR screening approaches to identify gene targets in thousands of single‐cells, using panels of guide RNAs.^57^

Nanowell approaches, such as Seq‐Well^51^ in which cells are partitioned into individual nanowells with uniquely barcoded oligo‐dT sequences offer similar levels of throughput but without the need to for microfluidics, reducing the overall cost and increasing the portability of the system. These advances, and the availability of commercial variations of these approaches have become mainstream, enabling single‐cell transcriptomics studies to be performed in a diverse array of organs and organisms, at a scale where “whole‐organism” single‐cell sequencing has become feasible.^58^ Indeed, a combinatorial indexing approach for transcriptomic analysis (sci‐RNA‐seq) has been applied to sequence over 40 000 cells from L2 *Caenorhabditis elegans* larvae, representing over 50‐fold coverage of all of the cells in a single organism.^13^

Throughput in single‐cell transcriptomics experiments has reached astonishing levels, with experiments now detailing thousands to millions of cells now becoming routine. However, there is minimal change in the total amount of sequencing performed in a single experiment, and thus the transcriptional profiling of these large numbers of cells focusses on enumeration of 3′ tag sequences and shallow coverage of the whole transcriptome. The majority of single‐cell transcriptomics analysis uses 3′ tag sequencing approaches and assigns cell types as a result of clustering—for example, using principle components analysis (PCA) or *t*‐distributed stochastic neighbor embedding (*t*‐SNE) plots.^59^ Fortunately, cell type heterogeneity can readily be detected from as few as 50 000–100 000 sequencing reads per cell, thus, minimal sequencing of a single cell's transcriptome allows categorization of cell identity—and many thousands of cells can be profiled and classified in parallel.

The transcriptomes generated at this throughput are currently necessarily superficial, and nuanced changes in lower expressed genes cannot be detected. Furthermore, due to the 3′ selectivity of these methods, UTR sequences, alternative splicing, RNA editing, mutations, and allelic expression can only fractionally be considered. Similarly, small RNA molecules, including microRNAs will not be detected using these approaches, although protocols for plate‐based small RNA‐sequencing have been described.^60^ It is clear that these aspects of cellular identity are essential for the functioning of the cell and indeed the organism—thus, methods which explore the full diversity of transcript heterogeneity, albeit at much lower throughput, remain highly relevant. A hybrid approach between broad, shallow sequencing of a population of cells and focused lower‐throughput sequencing of a target population perhaps offers the best means to globally explore cellular heterogeneity while maintaining the ability to focus on specific cellular regulatory phenomena.

### 2.4 Single‐Cell Proteomics

The functional identity of a cell is largely a product of its proteome—it is through proteins and post‐translational modifications that cells sense and respond to virtually all extrinsic and intrinsic stimuli. A comprehensive overview of a cell's proteome would perhaps give the most detailed definition of cell type and state possible by molecular means, but the limitations of current approaches make such observations impossible. Neither antibody nor mass spectrometric–based detection or quantification of proteins has the throughput or sensitivity required for proteome‐wide screening. However, advances in antibody labeling and detection, microfluidics, and recently, sensitive mass spectrometry approaches, are beginning to show the potential of proteomic analysis of single cells, and protein level detection remains an important tool for validation of single‐cell RNA‐seq results.

Western blotting approaches sensitive enough to detect proteins from single cells have been developed.^61^ Such approaches may offer a unique specificity due to reporting of protein size as well as quantitation, which additionally may allow protein isoform and modification detection. Using micropatterned polyacrylamide arrays, this approach enables capture, lysis, and electrophoresis of ∼3000 individual cells in parallel, followed by cross‐linking to immobilize the protein and detection using primary and secondary antibodies. By stripping and reprobing the gel, detection of ten proteins in the same single cell was demonstrated. Advances in this method have even enabled subcellular fractionation of single cells to enable parallel analysis of protein expression in the nucleus and cytoplasm of the same cell.^62^

High‐throughput, multiplexed analysis of protein expression in single cells has been carried out by FACS analysis for several decades. The detection of immunofluorescently tagged proteins in thousands of single cells is routine for FACS analysis, with modern high parameter instruments capable of analyzing up to 50 parameters in parallel. This is, however, technically challenging due to the potential for overlap between fluorescent spectra and these high parameter applications likely represent the upper limit of the capability of FACS. The CyTOF approach is a variant of FACS in which antibodies are labeled with heavy metal ion tags rather than fluorophores; the abundance of each metal ion labeling the cell is read out using time‐of‐flight mass spectrometry.^63^ These instruments have over 100 non‐overlapping detection channels and thus high levels of multiplexing (>40 proteins in parallel) are possible. Both FACS and CyTOF have potential to measure extracellular and intracellular parameters, including phosphorylation events in fixed and permeabilized cells. Increases in multiplexing may be possible through the use of DNA barcode–tagged antibodies and reading out levels of protein abundance using NGS technology. The Abseq approach has demonstrated DNA‐barcoded antibodies, in parallel with a custom microfluidics platform, can be used to screen surface protein abundance in single cells with high‐throughput and a theoretically unlimited capacity for multiplexing.^64^

However, antibody‐based methods will always be dependent on the specificity and availability of the antibody. Unbiased proteomic analysis of single cells is challenging due to limitations on the sensitivity of mass spectrometry techniques, and the lack of a PCR‐like method to amplify protein signals. The phase‐enhanced sample preparation (SP3) method was described^65^ in which paramagnetic beads are used to enrich proteins or peptides from low‐input samples, including single human oocytes, generating input material for liquid chromatography coupled to tandem mass spectrometry (LC‐MSMS). From individual oocytes, which are atypically large cells (diameter >100 μm), as many as 450 proteins were detected. Work in single Xenopus blastomeres combining high‐resolution mass spectrometry with capillary electrophoresis and electrospray ionization^66^ or single‐cell reverse‐phased liquid chromatography‐electrospray ionization tandem mass spectrometry^67^ have further been able to identify several hundred proteins; though again, these cells are atypically large.

Recently, Single‐Cell ProtEomics by Mass Spectrometry (SCoPE‐MS)^68^ was developed and applied to cancer cell lines and differentiating ES cells, bringing the sensitivity of single‐cell proteomics down to a level where normal‐sized cells can be analyzed. The method, which utilizes tandem mass tagging (TMT) to enable peptides from up to eight single cells to be digested, individually labeled and pooled with 200 carrier cells to provide sufficient ions for peptide sequence identification. This enabled detection and quantification of over 500 proteins in Jurkat and U937 cells (diameter ∼11 μm) and over 1000 proteins in mouse embryonic stem (ES) cells. These data were sufficient to perform differential clustering of individual cells, and to identify differential protein expression signatures between cell types.

Microfluidics technologies constitute another class of tools available to collect highly multiplexed measurements of proteins from single cells. Microchip‐based proteomics analysis enable simultaneous quantification of up to 40 nuclear, cytoplasmic, membrane and secreted proteins across thousands of single cells, with the sensitivity threshold of as low as a few hundred protein copies per cell.^69^ In particular, and in contrast to CyTOF, these tools allow measurement of secreted proteins from viable cells and offer control over the cell's microenvironment before analysis, allowing functional screens to be performed.^70^

A “microengraving” approach using small volume microwells in an array format can be used to isolate and culture single cells. Using an antibody‐coated substrate to cap the microwell array and capture secreted proteins, an ELISA immunoassay can be performed to enable protein quantification.^71^ A different approach used immobilized lipid bilayers and tethered ligands on the surface arrays of subnanoliter wells. These single‐cell lipid bilayer‐tethered ligands on arrays have enabled functional single‐cell analysis of T‐cell activation characterizing cytokines secreted from activated human T cell clones.^72^

A related approach is the single‐cell barcode chips (SCBCs) that patterns a capture antibody array in a single‐cell microwell so that different proteins can be detected at designated array spots. Cells are lysed on‐chip, and the levels of released proteins are assayed using the antibody arrays. SCBC data have been used to examine altered signal transduction networks in tumor and immune cells.^73^

### 2.5 Single‐Cell Metabolomics

The metabolome, defined as the full collection of all low‐molecular‐weight metabolites that are produced by a cell, could be a key indicator of cell state—reflecting the precise metabolic activity and condition within the cell. However, the metabolome is challenging to measure at the single‐cell level, largely due to the diversity and rapid dynamics of the system, coupled with the lack tagging and/or amplification approaches for small molecules.^74^

Advances in optical tools (such as genetically encoded optical nanosensors) together with improved expression systems and in vivo imaging have made possible the measurement of metabolites in real time and in single cells.^74^, ^75^ Single‐cell mass spectrometry has empowered, as well, metabolomic investigations to the size of individual cells and subcellular structures. Using single‐cell capillary electrophoresis coupled to electrospray ionization time‐of‐flight MS, metabolites quantification has been performed on individual isolated neurons,^76^ and analytical validation of a single‐cell metabolite analysis using the microarrays for mass spectrometry (MAMS) platform has also been applied to monitor cellular responses upon environmental and genetic perturbation.^77^

Given these developments, and the significance of cell metabolism in the definition cell state and function, it is anticipated that further technical advances will lead to more complete coverage of the metabolome, accurate and fast metabolites identification, and nondestructive measurement in single cells.

### 2.6 Multi‐omic Profiling of Single Cells

Cellular identity does not materialize from the isolated activities of the genome, epigenome, transcriptome, or proteome, but rather as a property of the interaction of each of these aspects with each other and other aspects of cell biology. As such, there is a considerable interest in capturing as much information from a single cell as possible—which has led to the emergence of single‐cell “multi‐omics” approaches (reviewed in detail in ref. 78).

Methods such as G&T‐seq^4^ and DR‐seq^79^ demonstrated the potential of parallel screening of the genomes and transcriptomes of single cells, by linking genomic diversity—aneuploidy, inter‐chromosomal fusions and SNVs—with transcriptional heterogeneity and the detection of expressed fusion transcripts and SNVs. Both methods demonstrated a correlation between chromosomal copy number and gene expression, while the scalable nature of the G&T‐seq protocol enabled focused analysis of a parallel inter‐chromosomal fusion and its resultant fusion transcript, as well as integration of SNV information between genomes and transcriptomes.^4^ We and others are now applying these techniques in the study of primary cancer cells, as well as rare circulating tumor cells, with the aim of extracting a more complete molecular profile of cellular evolution and its functional/phenotypic consequences in cancer.

Linking epigenomic and transcriptomic measurements of the same single cell allows exploration of the regulatory mechanisms underlying transcriptional heterogeneity. Further development of the G&T‐seq protocol has enabled parallel methylation profiling and transcriptional analysis of single cells (scM&T‐seq^26^). Similarly, scTrio‐Seq integrates parallel transcriptome analysis and bisulfite sequencing DNA copy number analysis to perform genome, epigenome, and transcriptome analysis of the same cell.^80^ Recently, the G&T‐seq method has further been adapted to incorporate NOME‐seq^34^ analysis of the same single cell, giving a triple readout of chromatin accessibility, DNA methylation, and gene expression.^81^

Perhaps most interesting for understanding dynamic, rapid cell type, and state transitions is the integration of transcriptomic and proteomic information. Several methods allowing such observations have emerged, though all are currently dependent on antibodies which “translate” the protein signal into a nucleic acid signature which can be read out by qPCR^82^ or sequencing.^83^ Proximity extension assays (PEA) and proximity ligation assays (PLA) involve the conjugation of nucleic acid “barcodes” to antibody pairs which recognize different epitopes of the same protein (or protein complex) and protein abundance can subsequently be measured by qPCR‐based detection,^82^ in parallel with transcriptomic measurements from the same single cell.

Recently, microfluidics approaches for single‐cell mRNA‐seq have been coupled with oligonucleotide tagged antibodies to enable highly multiplexed protein analysis and parallel transcriptome analysis. The cellular indexing of transcriptomes and epitopes (CITE‐seq) approach labels cells with antibodies tagged with barcode sequences flanked by polyadenine and PCR handle sequences compatible with transcriptome‐wide amplification.^83^ Thus, when standard whole transcriptome amplification is performed using a drop‐seq or 10X Chromium platform, the antibody barcodes are simultaneously amplified, enabling parallel transcript and protein quantification. Such approaches have the potential for high levels of multiplexing as, unlike fluorescence or mass cytometry‐based detection, there is a virtually unlimited number of barcodes or tags that can be used in parallel.

Integration of metabolite assays with proteomics assays might directly resolve connections between protein signaling networks and functional small molecule metabolites within the cell, reflecting the cell's state and type. The single‐cell barcode chip (SCBC) platform described above can be used to integrate quantitative measurements for intracellular metabolites with functional protein immunoassays into a microarray format.^84^

Approaches for multi‐omic analysis of single cells continue to develop at pace, and as methods improve and sequencing throughput increases, it seems likely that methods to capture as much as possible of a single cell's identity will become increasingly important in focused studies of cellular heterogeneity.

## 3 Applications of Single‐Cell Technology in Biomedical Research and Basic Biology

Single‐cell transcriptomic studies have been widely applied to “atlassing” studies of tissues, organs, and even whole organisms.^13^ Indeed, the Human Cell Atlas, which aims to characterize every cell type and state in the human body, has emerged as one of the most ambitious, large‐scale projects in biology since the Human Genome Project.^85^, ^86^ Such studies will inevitably have profound impact on our understanding of cellular heterogeneity and the role it plays in the division of labor within the human body, during normal development and disease. However, single‐cell technologies are also highly applicable in focused areas of research, and are set to become commonplace tools in a diverse array of fields, notably in clinical and biomedical research, but also in studies of plant and microbial biology.

### 3.1 Single‐Cell Analysis in Stem Cell Biology

Stem cells are characterized by both being capable of unlimited self‐renewal and having the potential to differentiate into specialized types of cells,^87^ and understanding how they establish their molecular identity has profound implications for developmental biology and regenerative medicine. Both embryonic and adult tissue stem cells represent a rare but heterogenous population, being composed by a mixture of intermediate and differentiated cell types, subtypes, and states. Performing omics analysis on bulk stem cell populations will hide this intrinsic heterogeneity, and single‐cell approaches are uniquely able to identify cell‐specific phenotypes and cell‐to‐cell variation in state. Single‐cell RNA‐seq analysis has revealed that transcriptional regulators and genes associated with pluripotency have variable expression among individual cell from human and mouse embryos^88^ and embryonic stem cell (ESCs)^89^ and has identified ESC subpopulations showing distinct transcriptional profiles.^48^, ^90^ Single‐cell RNA‐seq methods have also been applied to investigate tissue‐specific stem cell populations, and as a result, novel stem cell types have been identified and a deeper understanding of the transcriptome dynamics of developmental process under physiological and perturbed conditions has been provided.^91^, ^92^

Application of single‐cell methods to investigate the hematopoietic system has led to paradigm shifts in our understanding of cellular heterogeneity in hematopoiesis and how this is disrupted in disease.^3^, ^53^, ^92^, ^93^ Observations from single‐cell functional assays, and gene expression profile analysis have provided evidence of considerable functional heterogeneity, self‐renewal, and lineage potentials, even within the most stringently defined HSC populations.^3^, ^94^ Another exciting single‐cell analysis approaches applied to hematopoiesis is the ability to measure multiple proteins expressed by single cells through mass cytometry. This technique can also be combined with transcriptional measurements, and can be used as a highly multiplexed imaging platform that could be applied to study the HSC niche.^95^ Finally, single‐cell epigenetic analysis is likely to become of particular interest in the study of HSCs as epigenetic regulation appears to play a major role in the functional lineage biases of HSCs. Single‐cell technologies, including transcriptomic, proteomics, and epigenomic analysis, will enable molecular dissection of this heterogeneity and the regulation and maintenance of hematopoiesis in health and disease.

### 3.2 Single‐Cell Analysis in Cancer

Cancer is one of the most common manifestations of genomic mosaicism in humans. The study of tumor heterogeneity is further complicated by their often polyclonal nature, with single‐cell derived clones harboring genetic and epigenetic alterations that differ from the host genome and from other cells within the tumor. Although the genetic heterogeneity and the evolutionary principles governing resistance are actively being discovered, tools enabling the study of molecular processes that govern tumor progression are lacking. The single cell is the fundamental substrate upon which mutational mechanisms and the principles of selection act to evolve the complex structure that is a tumor mass.^96^ Thus, understanding single cancer cells at their individual level and as an interacting, dynamic system, will undoubtedly advance our understanding of all facets of tumor biology and eventually therapeutic resistance.^97^

Single‐cell technologies able to characterize a single‐cell genome, transcriptome, epigenome, and proteome could provide a clearer picture of tumor biology complexity at every phase of tumor development, with potential applications in new therapeutic approaches, cancer treatment, and clinical management. The application of bulk exome sequencing, targeted deep‐sequencing, and parallel single‐cell DNA sequencing to study clonal evolution during metastatic dissemination in two colon cancer patients, demonstrated that, in contrast to bulk sequencing methods, single‐cell analysis was able to distinguish between a tumor self‐seeding later dissemination and an early dissemination models of metastasis. Moreover, single‐cell sequencing identified a rare ancestral subpopulation, composed of three diploid cells carrying a mutation in the APC gene that initiated the tumorigenesis and subsequently gave rise to the primary tumor and liver metastasis.^98^ Using a nanogrid‐based single‐nucleus RNA‐seq system, Gao et al. compared transcriptional profiles of cancer nuclei and cancer cells and to study phenotypic diversity and subpopulations in breast cancer frozen samples.^99^ They showed that nuclear transcriptomes are representative of whole cellular transcriptomes and were able to identify co‐existence of multiple breast cancer subtypes and a minor subpopulation of highly proliferative cancer cells within the same patient's tumor.

Single‐cell sequencing can also be applied to the molecular phenotyping of circulating tumor cells (CTCs),^100^, ^101^ circulating rare cancer cells heralding tumor metastasis. As CTCs can be collected in a minimally invasive procedure through a conventional blood sample single‐cell genome sequencing of CTCs could provide an attractive surrogate biopsy of primary or metastatic tumors. The single‐cell molecular analysis of circulating tumor cells has already confirmed the high degree of heterogeneity of intracellular population within the same patients and across different patients, and has identified the coexistence of different drug‐resistance mechanisms in refractory tumors.^101^ Single CTCs from lung cancer patients displayed characteristic cancer‐associated SNVs and indel profiles in exomes of CTCs that were varying from cell to cell,^102^ demonstrating the feasibility of an approach that could be widely applied in the study of cancer metastasis.

### 3.3 Single‐Cell Analysis in Reproductive Medicine

Novel preimplantation genetic screening (PGS) for chromosomal abnormalities has recently been developed to improve clinical outcomes in patients undergoing in vitro fertilization (IVF) as aneuploidy in one of the most prevalent genetic abnormality in human embryos. Fluorescence in situ hybridization (FISH)–based screening is unable to provide a comprehensive analysis of all chromosomes, while the development of WGA technologies has enabled the analysis of whole chromosomes aneuploidies in single cells. Performing WGA on oocytes, one of the blastomeric cells from day‐3 embryos, or from trophectoderm cells from day‐5 blastocyst embryos, enables comprehensive chromosome analyses on various genome analytical platforms, such as a comparative genomic hybridization array^103^ single‐nucleotide polymorphism array^104^ or multiplex quantitative PCR analysis.^105^ The rapid development of NGS and advancement of WGA techniques will enable use of this technologies in clinical practice, as demonstrated by the chromosomal and mitochondrial genome copy number profiling in human IVF embryos using the MALBAC WGA approach.^106^

### 3.4 Applications of Single‐Cell Technology in Plant Research

Examples of plant single‐cell analysis are relatively uncommon, although the technology has a number of exciting potential applications. As with animal and human samples, genotyping, developmental studies, and cell‐typing using single‐cell approaches are all highly relevant, as is the use of these approaches for the analysis of biomolecule synthesis and interactions.^107^ A major challenge in plant single‐cell analysis is the presence of a cell wall, and protocols for rapid tissue dissociation and a common cell wall lysis method are lacking. Indeed, with the exception of pollen, few cell types in multicellular plants can be readily dissociated without enzymatic treatment, and removal of the cell wall has consequences for the stability of the remaining cell protoplast, as well as potential repercussions for gene expression in the cell due to the level of stress caused by enzymatic or mechanical cell wall digestion.^108^ However, single‐cell transcriptome amplification approaches are compatible with protoplast amplification, and studies of cell identity in *Arabidopsis thaliana* have been successfully performed.^109^

Single‐cell genome sequencing may have immediate and highly beneficial application in pollen typing, applicable in both basic molecular genetics and agricultural breeding. During the meiotic cycle, chromatids recombine resulting genetic differences in each of the daughter cells. The frequency of segregation of different alleles into different pollen grains then determines the genetic diversity and distribution of beneficial traits (e.g., crop yield) of the offspring plants. Currently, studies of plant population genomics are performed using low‐throughput cytological assessment of the pollen grains and conventional breeding, with large numbers of offspring plants needed per study. Often these plants have long generational times, for example, wheat can take up to 9 months to mature in the field, making the process slow and costly.

By sequencing the genomes of single pollen grains, it may be possible to haplotype the parental chromosomal contribution and understand factors regulating the frequency of crossing‐over, and thus population genetic diversity. Pollen‐typing has advantages which work to help with some of these issues. It is high‐throughput, often using FACS, and only one plant is needed for studies such as those looking at quantitative‐trait loci (QTL) association or mapping which usually require thousands of replicates.^110^ Dreissig et al. studied barley (*Hordeum vulgare*) pollen in order to assess the number and location of recombination sites along the length of each chromosome, testing the cytological hypothesis that the majority of the sites are located at the distal ends despite the “peri‐centromeric” regions.^111^ Single‐cell multi‐omic approaches may further enable researchers to link this whole genome sequencing with other “omic” data such as those from the transcriptome, methylome or proteome to further understand the biology of plant meiosis and pollen formation.

### 3.5 Single‐Cell Analysis of Microbial Communities

Since microbial populations are often complex and consist of a community of multiple species, single‐cell analysis may be vital in dissecting molecular heterogeneity between cells.^112^ The applications are wide‐ranging from deciphering phylogenetic trees and evolutionary mechanisms, to discovering novel metabolic features within a microbiome,^113^ monitoring and optimizing the productivity in industrial bioprocesses,^114^ showing the diversity of symbiotic interactions as well as viral integrations, to elucidating “rare biospheres.”^112^ Currently, microbiomes are predominantly categorized by sequencing of the small ribosomal 16S subunit using targeted primers. This targeted technique only amplifies a small proportion of the entire genomic content within a microbial cell, often misses less efficiently amplified rare cells within a population and cannot amplify certain members of the *Actinobacteria* and *Crenarchaeota*.^112^ Adapting existing eukaryote single‐cell approaches for prokaryotes is technically challenging, due to difficulties in sorting single microbial cells, the lack of a cell lysis method which can be applied across all taxa, WGA biases and variability in genomes within a population, and single‐cell sequencing or analysis in general within the microbial field is relatively uncommon. However, considerable effort is being made to resolve these issues, and instruments specifically designed for microbial sorting or microfluidic processing^22^ are emerging, as well as techniques to improve the already existing tools. WGA‐X, an improvement of the already existing genome amplification enzyme phi29, helps with environmental and viral samples with high GC content.^115^ Recently, a microfluidic platform for single‐cell compartmentalization and WGA of microbial communities (SiC‐seq) was described, enabling genomic processing of over 15 000 single cells, including those collected from marine water samples.^22^ Again, using shallow sequencing of each cell, the method allows screening of bacterial populations for anti‐microbial resistance (AMR) genes, virulence factors and mobile genetic elements (e.g., phage). The diversity inherent in real‐world bacterial communities make them a fertile ground for the application of single‐cell approaches, particularly in the understanding of population evolution and the development of traits such AMR.

## 4 Future Perspectives/Outlook

Approaches for the study of the molecular identity of single cells have emerged and been adapted at a rapid pace over the last 5 years. Through application in large scale, multi‐center studies of whole organism biology, such as the Human Cell Atlas,^86^ and more focused studies of discreet biological cell types and states, these techniques—in particular, single‐cell transcriptomics—are becoming routine tools in cellular genomics. Continued technical improvement, adoption, and adaptation of techniques will see further uptake of the methods in plant and microbial research.

However, continued technical development is essential to maximize the amount of information that can be retrieved from a single cell. Each of the methods described in this review has limitations, particularly in the coverage they provide of the analyte of interest, which is particularly important where base‐level events (e.g., SNVs or individual base modifications) are to be considered. Improvements in molecular biology and microfluidics may resolve some of these issues, and computational approaches for imputation of missing data are also increasingly being applied.^116^ As sequencing capacity increases, both in terms of yield and read length, tools for high‐throughput single‐cell splice variant analysis will emerge, and be further integrated with genomic, epigenomic, and proteomic data from the same single cell. Methods which retain spatial information about the arrangement of cells within a tissue will be critical to resolving the contribution of physical interactions to the formation and function of biological structures.^95^, ^117^ Through the integration of spatiotemporal omics datasets from the same cells, it may be possible to construct detailed models of how cells establish, maintain, and change their identities throughout life.

## Conflict of Interest

The authors declare no conflict of interest.
